# 2-{[5-(Pyridin-4-yl)-4-*p*-tolyl-4*H*-1,2,4-triazol-3-yl]meth­yl}acrylic acid hemi­hydrate

**DOI:** 10.1107/S1600536813034077

**Published:** 2013-12-24

**Authors:** Renata Paprocka, Bożena Modzelewska-Banachiewicz, Andrzej K. Gzella

**Affiliations:** a Department of Organic Chemistry, Ludwik Rydygier Collegium Medicum in Bydgoszcz, Nicolaus Copernicus University in Torun, ul. A. Jurasza 2, 85-089 Bydgoszcz, Poland; b Department of Organic Chemistry, Poznan University of Medical Sciences, ul. Grunwaldzka 6, 60-780 Poznań, Poland

## Abstract

The asymmetric unit of the title compound, 2C_18_H_16_N_4_O_2_·H_2_O, consists of two organic molecules and one solvent molecule. The symmetry-independent organic mol­ecules have slightly different conformations: the 1,2,4-triazole ring forms dihedral angles of 84.61 (4), 89.68 (5) and 22.38 (6)°, respectively, with the 2-propenecarbocylic, *p*-tolyl and 4-pyridyl groups in one independent molecule, and 71.35 (4), 82.13 (5) and 24.82 (6)°, respectively, in the second. In the crystal, mol­ecules ralated by the 2_1_ screw axes are assembled *via* O—H⋯N and O—H⋯O hydrogen bonds into infinite chains and these are linked by further O—H⋯N hydrogen bonds into undulating sheets parallel to the *bc* plane. Adjacent sheets are connected by weak C—H⋯O inter­actions, forming a three-dimensional structure.

## Related literature   

For the pharmacological activity of 1,2,4-triazole derivatives, see: Amir & Shikha (2004 ▶); El-Serwy *et al.* (2013 ▶); McDowell *et al.* (2010 ▶); Modzelewska-Banachiewicz, Paprocka *et al.* (2012 ▶); Modzelewska-Banachiewicz, Ucherek *et al.* (2012 ▶); Siddiqui & Ahsan (2010 ▶); Sztanke *et al.* (2008 ▶); Wang *et al.* (2000 ▶).
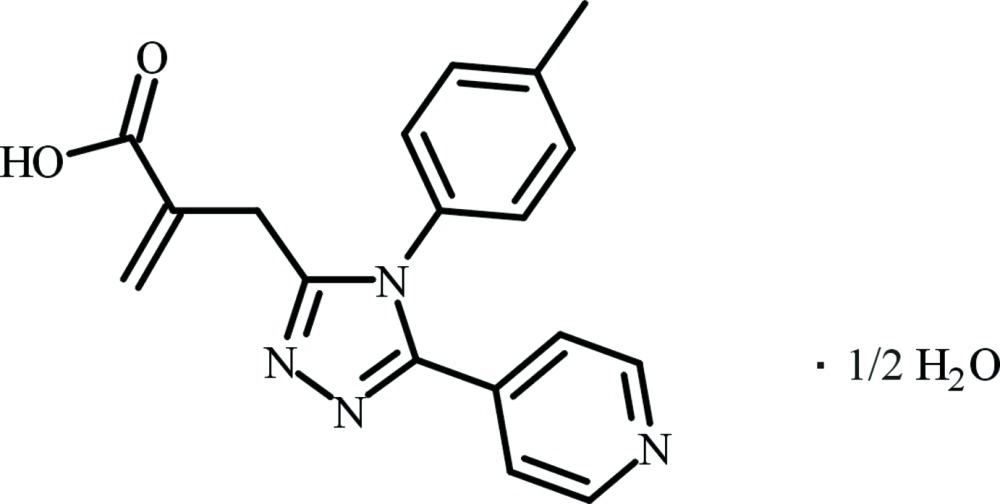


## Experimental   

### 

#### Crystal data  

2C_18_H_16_N_4_O_2_·H_2_O
*M* *_r_* = 658.71
Monoclinic, 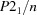

*a* = 10.0344 (1) Å
*b* = 16.1485 (2) Å
*c* = 20.1650 (3) Å
β = 98.699 (1)°
*V* = 3229.96 (7) Å^3^
*Z* = 4
Mo *K*α radiation
μ = 0.09 mm^−1^
*T* = 130 K
0.55 × 0.30 × 0.10 mm

#### Data collection  

Agilent Xcalibur Atlas diffractometer
Absorption correction: multi-scan (*CrysAlis PRO*; Agilent, 2011 ▶) *T*_min_ = 0.991, *T*_max_ = 1.000
22090 measured reflections
7724 independent reflections
6542 reflections with *I* > 2σ(*I*)
*R*_int_ = 0.015

#### Refinement  

*R*[*F* ^2^ > 2σ(*F* ^2^)] = 0.044
*wR*(*F* ^2^) = 0.119
*S* = 1.03
7724 reflections
460 parameters
H atoms treated by a mixture of independent and constrained refinement
Δρ_max_ = 0.80 e Å^−3^
Δρ_min_ = −0.28 e Å^−3^

### 

Data collection: *CrysAlis PRO* (Agilent, 2011 ▶); cell refinement: *CrysAlis PRO*; data reduction: *CrysAlis PRO*; program(s) used to solve structure: *SHELXS97* (Sheldrick, 2008 ▶); program(s) used to refine structure: *SHELXL97* (Sheldrick, 2008 ▶); molecular graphics: *ORTEP-3 for Windows* (Farrugia, 2012 ▶); software used to prepare material for publication: *WinGX* (Farrugia, 2012 ▶) and *PLATON* (Spek, 2009 ▶).

## Supplementary Material

Crystal structure: contains datablock(s) I, publication_text. DOI: 10.1107/S1600536813034077/bt6951sup1.cif

Structure factors: contains datablock(s) I. DOI: 10.1107/S1600536813034077/bt6951Isup2.hkl

Supporting information file. DOI: 10.1107/S1600536813034077/bt6951Isup3.cml

Additional supporting information:  crystallographic information; 3D view; checkCIF report

## Figures and Tables

**Table 1:** Hydrogen-bond geometry (Å, °)

*D*—H⋯*A*	*D*—H	H⋯*A*	*D*⋯*A*	*D*—H⋯*A*
O11*A*—H11*A*⋯N22*A* ^i^	0.92 (3)	1.77 (3)	2.6838 (17)	178 (2)
O11*B*—H11*B*⋯O25	0.98 (2)	1.59 (2)	2.5619 (16)	174 (2)
O25—H25*A*⋯N1*A*	0.86 (2)	2.00 (2)	2.8373 (17)	166 (2)
O25—H25*B*⋯N22*B* ^ii^	0.94 (2)	1.87 (2)	2.8050 (18)	171 (2)
C6*A*—H6*A*2⋯O10*B* ^iii^	0.97	2.51	3.3300 (18)	142
C24*A*—H24*A*⋯O10*B* ^iv^	0.93	2.41	3.2683 (18)	153
C24*B*—H24*B*⋯O10*A* ^iii^	0.93	2.57	3.4628 (18)	162

Symmetry codes: (i) 
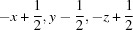
; (ii) 
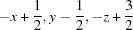
; (iii) 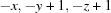; (iv) 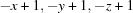.

**Fig. 1.**
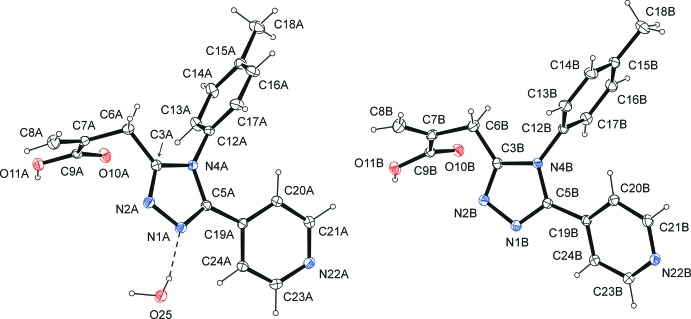
The independent molecules of (I) and water showing the atomic labelling scheme. Non-H atoms are drawn as 30% probability displacement ellipsoids and H atoms are drawn as spheres of an arbitrary radius.

**Fig. 2.**
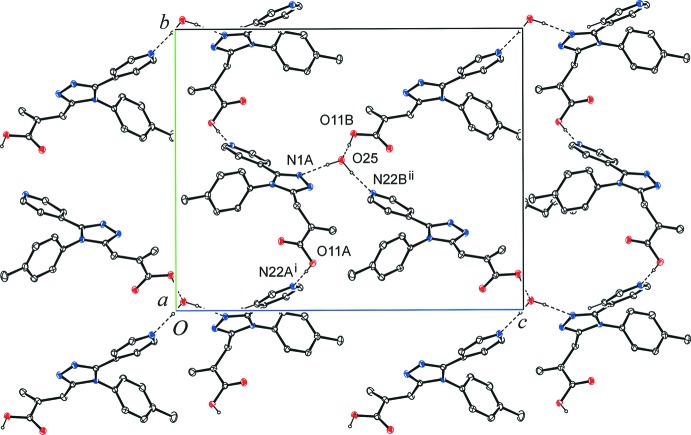
The hydrogen bonding in the title structure. For symmetry codes, see Table 1. H atoms not involved in hydrogen-bonding have been omitted for clarity.

**Fig. 3.**
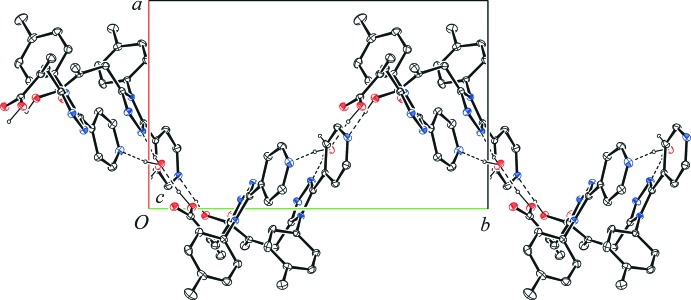
The hydrogen-bonded undulating sheet in (I).

## supplementary crystallographic information

### 1. Comment

The increasing diversity of small molecule libraries is an important source for the discovery of new drug candidates. In terms of this trend, triazole heterocycles are of importance in modern medicinal chemistry. 1,2,4-Triazole derivatives have been widely investigated for a range of pharmacological activities, such as anticancer (Sztanke *et al.*, 2008), antibacterial (Sztanke *et al.*, 2008), antiviral (McDowell *et al.*, 2010), antifungal (Wang *et al.*, 2000), anti-inflammatory (El-Serwy *et al.*, 2013), analgesic (Amir & Shikha, 2004), anticonvulsant (Siddiqui & Ahsan, 2010). Recently it was communicated that new 1,2,4-triazole-containing analogues of alkenoic acids showed antimicrobial activity (Modzelewska-Banachiewicz, Paprocka *et al.*, 2012). A series of 4,5-diarylsubstituted 1,2,4-triazole derivatives were also described as antiviral, antibacterial and anti-inflammatory agents (Modzelewska-Banachiewicz, Ucherek *et al.*, 2012).

The structure investigation of the title compound with potential antibacterial activity has been undertaken to determine its spatial structure and to facilitate the interpretation of ^1^H–, ^13^C-NMR and MS data.

The X-ray analysis showed that the crystal structure is a hemihydrate. The asymmetric part of the unit cell contains two symmetry-independent molecules, denoted A and B, of the compound (I) (solute) and one molecule of water (solvent) (Fig. 1). The independent molecules of (I) differ to a rather moderate extent in conformation. The weighted r.m.s. deviation for the superposition of the non-H atoms in both molecules is 0.674 Å (Spek, 2009). The differences concern the angular arrangement of the system of 1,2,4-triazole, towards three substituents, *i.e.* the 2-propenecarbocylic, *p*-tolyl and 4-pyridyl groups [molecule A: 84.61 (4), 89.68 (5) and 22.38 (6)°; molecule B: 71.35 (4), 82.13 (5) and 24.82 (6)°]. Angular orientation of the 2-propenecarbocylic fragment in the molecules A and B reveal two torsional angles N2—C3—C6—C7 and C3—C6—C7—C9 [molecule A: -47.08 (19) and -64.30 (15)°; molecule B: -7.6 (2) and -67.30 (17)°]. The first one indicates that in molecule A the N2—C3 bond adopts conformation halfway between synperiplanar and synclinal with respect to C6—C7 bond while in molecule B the mentioned bonds are synperiplanar to each other. The second torsional angle reveals mutual anticlinal orientation of the bonds C3—C6 and C7—C9. Conjugated system of double bonds C7═C8 and C9═O10 has *s-trans* conformation [torsion angle C8—C7—C9—O10: -175.09 (15)° (molecule A), -158.02 (15)° (molecule B)].

The interatomic distances C7═C8 take the values of 1.325 (2) in the molecule A and 1.317 (2) Å in the molecule B and confirm the presence of the double bond between these atoms.

In the crystal lattice, the symmetry-independent molecules A and B of (I) are connected with hydrogen bonds forming chains made separately from molecules A and B. Molecules A are joined to one another through the O11A—H11A···N22A^i^ hydrogen bonds while molecules B through the O11B—H11B···O25 and O25—H25B···N22B^ii^ hydrogen bonds. The latter are connected *via* water molecules (Table 1, Fig. 2). The neighbouring chains of molecules A and B are linked with O25—H25A···N1A hydrogen bonds into undulating sheets parallel to *bc* plane (Fig. 3). Moreover, in the crystal weak hydrogen bonds C6A—H6A2···O10B^iii^, C24A—H24A···O10B^iv^, C24B—H24B···O10A^iii^ are observed. They connect the adjacent sheets into three-dimensional structure.

### 2. Experimental

2-{[5-(Pyridine-4-yl)-4-*p*-tolyl-4*H*-1,2,4-triazol-3-yl]methyl}acrylic acid was obtained in reaction of *N*-*p*-tolylpyridine-4-carbothioamide with itaconic anhydride in the medium of anhydrous diethyl ether. Crystals were obtained after crystallization from water.

### 3. Refinement

The positions of the carboxyl groups and water H atoms were obtained from a difference Fourier map and were refined freely. The remaining H atoms were positioned geometrically and were refined within the riding model approximation: C_methyl_—H = 0.96 Å, C_methylene_—H = 0.97 Å, C(*sp*^2^)—H = 0.93 Å; *U*_iso_(H) = 1.2*U*_eq_(C) or 1.5*U*_eq_(C) for methyl H. The methyl groups were refined as rigid groups which were allowed to rotate. The difference density of 0.80 e / Å has no physical meaning and is rather due to the crystal quality.

### Crystal data

**Table:**

2C_18_H_16_N_4_O_2_·H_2_O	*F*(000) = 1384
*M**_r_* = 658.71	*D*_x_ = 1.355 Mg m^−^^3^
Monoclinic, *P*2_1_/*n*	Melting point = 416–418 K
Hall symbol: -P 2yn	Mo *K*α radiation, λ = 0.71073 Å
*a* = 10.0344 (1) Å	Cell parameters from 9224 reflections
*b* = 16.1485 (2) Å	θ = 2.0–29.1°
*c* = 20.1650 (3) Å	µ = 0.09 mm^−^^1^
β = 98.699 (1)°	*T* = 130 K
*V* = 3229.96 (7) Å^3^	Lath, colourless
*Z* = 4	0.55 × 0.30 × 0.10 mm

### Data collection

**Table:**

Agilent Xcalibur Atlas diffractometer	7724 independent reflections
Radiation source: Enhance (Mo) X-ray Source	6542 reflections with *I* > 2σ(*I*)
Graphite monochromator	*R*_int_ = 0.015
Detector resolution: 10.3088 pixels mm^-1^	θ_max_ = 29.2°, θ_min_ = 2.2°
ω scans	*h* = −13→12
Absorption correction: multi-scan (*CrysAlis PRO*; Agilent, 2011)	*k* = −18→21
*T*_min_ = 0.991, *T*_max_ = 1.000	*l* = −26→27
22090 measured reflections	

### Refinement

**Table:**

Refinement on *F*^2^	Primary atom site location: structure-invariant direct methods
Least-squares matrix: full	Secondary atom site location: difference Fourier map
*R*[*F*^2^ > 2σ(*F*^2^)] = 0.044	Hydrogen site location: inferred from neighbouring sites
*wR*(*F*^2^) = 0.119	H atoms treated by a mixture of independent and constrained refinement
*S* = 1.03	*w* = 1/[σ^2^(*F*_o_^2^) + (0.0533*P*)^2^ + 1.7812*P*] where *P* = (*F*_o_^2^ + 2*F*_c_^2^)/3
7724 reflections	(Δ/σ)_max_ < 0.001
460 parameters	Δρ_max_ = 0.80 e Å^−^^3^
0 restraints	Δρ_min_ = −0.28 e Å^−^^3^

### Special details

**Table:**

Experimental. none
Geometry. All s.u.'s (except the s.u. in the dihedral angle between two l.s. planes) are estimated using the full covariance matrix. The cell s.u.'s are taken into account individually in the estimation of s.u.'s in distances, angles and torsion angles; correlations between s.u.'s in cell parameters are only used when they are defined by crystal symmetry. An approximate (isotropic) treatment of cell s.u.'s is used for estimating s.u.'s involving l.s. planes.
Refinement. Refinement of *F*^2^ against ALL reflections. The weighted *R*-factor *wR* and goodness of fit *S* are based on *F*^2^, conventional *R*-factors *R* are based on *F*, with *F* set to zero for negative *F*^2^. The threshold expression of *F*^2^ > 2σ(*F*^2^) is used only for calculating *R*-factors(gt) *etc*. and is not relevant to the choice of reflections for refinement. *R*-factors based on *F*^2^ are statistically about twice as large as those based on *F*, and *R*- factors based on ALL data will be even larger.

### Fractional atomic coordinates and isotropic or equivalent isotropic displacement parameters (Å^2^)

**Table:**

	*x*	*y*	*z*	*U*_iso_*/*U*_eq_	
N1A	0.13167 (12)	0.47922 (8)	0.35710 (6)	0.0239 (3)	
N2A	0.03447 (12)	0.43871 (8)	0.38651 (6)	0.0246 (3)	
C3A	−0.06428 (14)	0.42016 (8)	0.33856 (7)	0.0210 (3)	
N4A	−0.03617 (11)	0.44814 (7)	0.27792 (6)	0.0194 (2)	
C5A	0.08908 (13)	0.48407 (8)	0.29241 (7)	0.0201 (3)	
C6A	−0.18670 (14)	0.37179 (9)	0.34822 (7)	0.0234 (3)	
H6A1	−0.2355	0.3558	0.3049	0.028*	
H6A2	−0.2457	0.4060	0.3706	0.028*	
C7A	−0.14745 (13)	0.29541 (9)	0.38961 (7)	0.0213 (3)	
C8A	−0.17641 (16)	0.28325 (11)	0.45081 (8)	0.0326 (3)	
H8A1	−0.1459	0.2358	0.4745	0.039*	
H8A2	−0.2272	0.3221	0.4701	0.039*	
C9A	−0.06644 (13)	0.23433 (9)	0.35751 (7)	0.0202 (3)	
O10A	−0.03092 (11)	0.24626 (7)	0.30337 (5)	0.0292 (2)	
O11A	−0.03137 (11)	0.16726 (7)	0.39398 (5)	0.0257 (2)	
H11A	0.033 (3)	0.1388 (16)	0.3757 (12)	0.067 (7)*	
C12A	−0.11815 (13)	0.43501 (8)	0.21379 (7)	0.0197 (3)	
C13A	−0.21393 (14)	0.49326 (9)	0.18935 (8)	0.0274 (3)	
H13A	−0.2255	0.5407	0.2140	0.033*	
C14A	−0.29256 (15)	0.48007 (10)	0.12756 (8)	0.0299 (3)	
H14A	−0.3558	0.5197	0.1104	0.036*	
C15A	−0.27859 (14)	0.40888 (10)	0.09086 (7)	0.0253 (3)	
C16A	−0.18208 (17)	0.35127 (10)	0.11710 (7)	0.0298 (3)	
H16A	−0.1715	0.3032	0.0931	0.036*	
C17A	−0.10138 (16)	0.36387 (9)	0.17820 (7)	0.0271 (3)	
H17A	−0.0368	0.3249	0.1950	0.033*	
C18A	−0.36508 (17)	0.39371 (12)	0.02439 (8)	0.0362 (4)	
H18A	−0.4299	0.4375	0.0153	0.054*	
H18B	−0.4113	0.3418	0.0257	0.054*	
H18C	−0.3094	0.3921	−0.0103	0.054*	
C19A	0.17137 (13)	0.51971 (8)	0.24532 (7)	0.0200 (3)	
C20A	0.12086 (14)	0.54991 (9)	0.18206 (7)	0.0252 (3)	
H20A	0.0289	0.5482	0.1664	0.030*	
C21A	0.20962 (15)	0.58253 (9)	0.14279 (8)	0.0271 (3)	
H21A	0.1746	0.6030	0.1006	0.033*	
N22A	0.34290 (12)	0.58630 (8)	0.16195 (6)	0.0254 (3)	
C23A	0.39050 (14)	0.55747 (9)	0.22296 (8)	0.0268 (3)	
H23A	0.4830	0.5600	0.2372	0.032*	
C24A	0.31073 (14)	0.52426 (9)	0.26586 (7)	0.0244 (3)	
H24A	0.3488	0.5051	0.3080	0.029*	
N1B	0.37740 (12)	0.80883 (8)	0.70883 (6)	0.0239 (3)	
N2B	0.45728 (12)	0.77341 (8)	0.66600 (6)	0.0246 (3)	
C3B	0.56239 (14)	0.74131 (9)	0.70340 (7)	0.0213 (3)	
N4B	0.55646 (11)	0.75461 (7)	0.77019 (6)	0.0203 (2)	
C5B	0.43786 (13)	0.79731 (8)	0.77051 (7)	0.0204 (3)	
C6B	0.67580 (14)	0.69625 (10)	0.67849 (7)	0.0259 (3)	
H6B1	0.6900	0.6435	0.7015	0.031*	
H6B2	0.7579	0.7284	0.6890	0.031*	
C7B	0.64739 (14)	0.68143 (9)	0.60410 (7)	0.0246 (3)	
C8B	0.72326 (16)	0.71225 (11)	0.56226 (9)	0.0337 (4)	
H8B1	0.7074	0.6975	0.5172	0.040*	
H8B2	0.7927	0.7487	0.5778	0.040*	
C9B	0.53499 (14)	0.62252 (9)	0.57998 (7)	0.0221 (3)	
O10B	0.48977 (10)	0.57518 (7)	0.61765 (5)	0.0273 (2)	
O11B	0.49295 (11)	0.62687 (7)	0.51487 (5)	0.0279 (2)	
H11B	0.419 (2)	0.5879 (14)	0.5013 (11)	0.053 (6)*	
C12B	0.64672 (14)	0.72110 (9)	0.82592 (7)	0.0206 (3)	
C13B	0.61305 (16)	0.64845 (9)	0.85523 (8)	0.0270 (3)	
H13B	0.5334	0.6210	0.8388	0.032*	
C14B	0.69968 (17)	0.61681 (10)	0.90969 (8)	0.0320 (3)	
H14B	0.6775	0.5676	0.9294	0.038*	
C15B	0.81822 (16)	0.65707 (11)	0.93523 (8)	0.0316 (3)	
C16B	0.84978 (16)	0.73014 (11)	0.90471 (8)	0.0325 (3)	
H16B	0.9288	0.7581	0.9214	0.039*	
C17B	0.76551 (15)	0.76198 (10)	0.84992 (8)	0.0280 (3)	
H17B	0.7885	0.8104	0.8294	0.034*	
C18B	0.9097 (2)	0.62366 (14)	0.99552 (9)	0.0495 (5)	
H18D	0.8716	0.5739	1.0107	0.074*	
H18E	0.9192	0.6642	1.0308	0.074*	
H18F	0.9966	0.6116	0.9834	0.074*	
C19B	0.38527 (14)	0.83183 (8)	0.82898 (7)	0.0207 (3)	
C20B	0.46609 (15)	0.85228 (9)	0.88900 (7)	0.0258 (3)	
H20B	0.5573	0.8390	0.8961	0.031*	
C21B	0.40785 (16)	0.89298 (10)	0.93822 (8)	0.0285 (3)	
H21B	0.4625	0.9062	0.9783	0.034*	
N22B	0.27817 (13)	0.91411 (8)	0.93119 (6)	0.0284 (3)	
C23B	0.20068 (15)	0.89272 (10)	0.87382 (8)	0.0281 (3)	
H23B	0.1097	0.9064	0.8684	0.034*	
C24B	0.24830 (15)	0.85145 (9)	0.82223 (7)	0.0249 (3)	
H24B	0.1901	0.8369	0.7836	0.030*	
O25	0.28894 (12)	0.53329 (7)	0.47820 (6)	0.0319 (3)	
H25A	0.254 (2)	0.5184 (13)	0.4385 (12)	0.045 (6)*	
H25B	0.274 (2)	0.4899 (16)	0.5073 (12)	0.064 (7)*	

### Atomic displacement parameters (Å^2^)

**Table:**

	*U*^11^	*U*^22^	*U*^33^	*U*^12^	*U*^13^	*U*^23^
N1A	0.0241 (6)	0.0267 (6)	0.0205 (6)	−0.0052 (5)	0.0021 (5)	−0.0002 (5)
N2A	0.0250 (6)	0.0275 (6)	0.0214 (6)	−0.0050 (5)	0.0034 (5)	0.0003 (5)
C3A	0.0222 (6)	0.0203 (6)	0.0206 (7)	0.0014 (5)	0.0038 (5)	0.0000 (5)
N4A	0.0187 (5)	0.0199 (5)	0.0192 (6)	−0.0007 (4)	0.0019 (4)	−0.0013 (4)
C5A	0.0202 (6)	0.0188 (6)	0.0208 (6)	−0.0008 (5)	0.0013 (5)	−0.0011 (5)
C6A	0.0194 (6)	0.0257 (7)	0.0253 (7)	0.0004 (5)	0.0044 (5)	0.0006 (6)
C7A	0.0171 (6)	0.0241 (7)	0.0228 (7)	−0.0034 (5)	0.0034 (5)	−0.0009 (5)
C8A	0.0334 (8)	0.0355 (9)	0.0308 (8)	0.0030 (7)	0.0110 (7)	0.0028 (7)
C9A	0.0175 (6)	0.0241 (7)	0.0180 (6)	−0.0030 (5)	−0.0010 (5)	−0.0010 (5)
O10A	0.0352 (6)	0.0332 (6)	0.0205 (5)	0.0071 (5)	0.0084 (4)	0.0026 (4)
O11A	0.0264 (5)	0.0267 (5)	0.0244 (5)	0.0035 (4)	0.0057 (4)	0.0039 (4)
C12A	0.0184 (6)	0.0211 (6)	0.0189 (6)	−0.0020 (5)	0.0011 (5)	0.0000 (5)
C13A	0.0241 (7)	0.0243 (7)	0.0321 (8)	0.0030 (6)	−0.0016 (6)	−0.0065 (6)
C14A	0.0235 (7)	0.0306 (8)	0.0330 (8)	0.0066 (6)	−0.0039 (6)	−0.0006 (7)
C15A	0.0235 (7)	0.0320 (8)	0.0199 (7)	−0.0034 (6)	0.0017 (5)	0.0013 (6)
C16A	0.0432 (9)	0.0254 (7)	0.0199 (7)	0.0043 (6)	0.0016 (6)	−0.0033 (6)
C17A	0.0345 (8)	0.0254 (7)	0.0207 (7)	0.0087 (6)	0.0016 (6)	0.0007 (6)
C18A	0.0346 (8)	0.0482 (10)	0.0234 (8)	0.0012 (7)	−0.0030 (6)	−0.0034 (7)
C19A	0.0213 (6)	0.0170 (6)	0.0215 (7)	−0.0012 (5)	0.0025 (5)	−0.0011 (5)
C20A	0.0201 (6)	0.0269 (7)	0.0277 (7)	0.0007 (5)	0.0011 (5)	0.0046 (6)
C21A	0.0268 (7)	0.0279 (7)	0.0258 (7)	−0.0003 (6)	0.0018 (6)	0.0062 (6)
N22A	0.0251 (6)	0.0246 (6)	0.0266 (6)	−0.0043 (5)	0.0045 (5)	0.0012 (5)
C23A	0.0203 (6)	0.0298 (8)	0.0293 (8)	−0.0039 (6)	0.0007 (6)	−0.0006 (6)
C24A	0.0245 (7)	0.0258 (7)	0.0215 (7)	−0.0033 (6)	−0.0012 (5)	0.0014 (6)
N1B	0.0249 (6)	0.0262 (6)	0.0203 (6)	0.0031 (5)	0.0028 (5)	−0.0024 (5)
N2B	0.0255 (6)	0.0275 (6)	0.0208 (6)	0.0030 (5)	0.0039 (5)	−0.0034 (5)
C3B	0.0232 (6)	0.0212 (7)	0.0191 (6)	−0.0021 (5)	0.0015 (5)	−0.0034 (5)
N4B	0.0207 (5)	0.0219 (6)	0.0178 (5)	0.0012 (4)	0.0013 (4)	−0.0014 (4)
C5B	0.0208 (6)	0.0198 (6)	0.0203 (6)	0.0006 (5)	0.0019 (5)	−0.0003 (5)
C6B	0.0222 (7)	0.0302 (7)	0.0249 (7)	−0.0009 (6)	0.0025 (5)	−0.0067 (6)
C7B	0.0217 (6)	0.0267 (7)	0.0254 (7)	0.0008 (5)	0.0036 (5)	−0.0060 (6)
C8B	0.0313 (8)	0.0373 (9)	0.0331 (8)	−0.0058 (7)	0.0072 (7)	−0.0080 (7)
C9B	0.0204 (6)	0.0244 (7)	0.0212 (7)	0.0028 (5)	0.0024 (5)	−0.0023 (5)
O10B	0.0262 (5)	0.0300 (6)	0.0249 (5)	−0.0029 (4)	0.0018 (4)	0.0022 (4)
O11B	0.0282 (5)	0.0326 (6)	0.0218 (5)	−0.0067 (5)	0.0001 (4)	−0.0004 (4)
C12B	0.0231 (6)	0.0214 (7)	0.0168 (6)	0.0038 (5)	0.0011 (5)	−0.0020 (5)
C13B	0.0294 (7)	0.0241 (7)	0.0273 (7)	−0.0013 (6)	0.0035 (6)	−0.0011 (6)
C14B	0.0410 (9)	0.0277 (8)	0.0279 (8)	0.0073 (7)	0.0072 (7)	0.0066 (6)
C15B	0.0352 (8)	0.0384 (9)	0.0205 (7)	0.0154 (7)	0.0020 (6)	−0.0020 (6)
C16B	0.0260 (7)	0.0380 (9)	0.0306 (8)	0.0034 (6)	−0.0051 (6)	−0.0062 (7)
C17B	0.0269 (7)	0.0268 (7)	0.0287 (8)	−0.0015 (6)	−0.0006 (6)	0.0001 (6)
C18B	0.0492 (11)	0.0654 (13)	0.0305 (9)	0.0223 (10)	−0.0048 (8)	0.0064 (9)
C19B	0.0252 (7)	0.0183 (6)	0.0189 (6)	0.0016 (5)	0.0043 (5)	0.0017 (5)
C20B	0.0255 (7)	0.0282 (7)	0.0231 (7)	0.0045 (6)	0.0018 (6)	−0.0021 (6)
C21B	0.0333 (8)	0.0309 (8)	0.0209 (7)	0.0031 (6)	0.0026 (6)	−0.0023 (6)
N22B	0.0345 (7)	0.0300 (7)	0.0219 (6)	0.0056 (5)	0.0078 (5)	0.0005 (5)
C23B	0.0266 (7)	0.0328 (8)	0.0257 (7)	0.0054 (6)	0.0068 (6)	0.0020 (6)
C24B	0.0260 (7)	0.0275 (7)	0.0210 (7)	0.0024 (6)	0.0027 (5)	0.0007 (6)
O25	0.0368 (6)	0.0364 (6)	0.0203 (5)	−0.0149 (5)	−0.0025 (5)	0.0010 (5)

### Geometric parameters (Å, º)

**Table:**

N1A—C5A	1.3121 (18)	N1B—N2B	1.3875 (16)
N1A—N2A	1.3806 (16)	N2B—C3B	1.3073 (19)
N2A—C3A	1.3102 (18)	C3B—N4B	1.3740 (17)
C3A—N4A	1.3722 (17)	C3B—C6B	1.4999 (19)
C3A—C6A	1.4932 (19)	N4B—C5B	1.3763 (17)
N4A—C5A	1.3750 (17)	N4B—C12B	1.4381 (17)
N4A—C12A	1.4396 (17)	C5B—C19B	1.4722 (19)
C5A—C19A	1.4670 (18)	C6B—C7B	1.503 (2)
C6A—C7A	1.508 (2)	C6B—H6B1	0.9700
C6A—H6A1	0.9700	C6B—H6B2	0.9700
C6A—H6A2	0.9700	C7B—C8B	1.317 (2)
C7A—C8A	1.325 (2)	C7B—C9B	1.499 (2)
C7A—C9A	1.4871 (19)	C8B—H8B1	0.9300
C8A—H8A1	0.9300	C8B—H8B2	0.9300
C8A—H8A2	0.9300	C9B—O10B	1.2132 (17)
C9A—O10A	1.2140 (17)	C9B—O11B	1.3187 (17)
C9A—O11A	1.3263 (17)	O11B—H11B	0.98 (2)
O11A—H11A	0.91 (3)	C12B—C13B	1.379 (2)
C12A—C17A	1.378 (2)	C12B—C17B	1.384 (2)
C12A—C13A	1.381 (2)	C13B—C14B	1.391 (2)
C13A—C14A	1.387 (2)	C13B—H13B	0.9300
C13A—H13A	0.9300	C14B—C15B	1.385 (2)
C14A—C15A	1.386 (2)	C14B—H14B	0.9300
C14A—H14A	0.9300	C15B—C16B	1.389 (2)
C15A—C16A	1.389 (2)	C15B—C18B	1.508 (2)
C15A—C18A	1.503 (2)	C16B—C17B	1.385 (2)
C16A—C17A	1.383 (2)	C16B—H16B	0.9300
C16A—H16A	0.9300	C17B—H17B	0.9300
C17A—H17A	0.9300	C18B—H18D	0.9600
C18A—H18A	0.9600	C18B—H18E	0.9600
C18A—H18B	0.9600	C18B—H18F	0.9600
C18A—H18C	0.9600	C19B—C20B	1.391 (2)
C19A—C20A	1.388 (2)	C19B—C24B	1.397 (2)
C19A—C24A	1.3991 (19)	C20B—C21B	1.391 (2)
C20A—C21A	1.382 (2)	C20B—H20B	0.9300
C20A—H20A	0.9300	C21B—N22B	1.332 (2)
C21A—N22A	1.3362 (19)	C21B—H21B	0.9300
C21A—H21A	0.9300	N22B—C23B	1.338 (2)
N22A—C23A	1.3344 (19)	C23B—C24B	1.380 (2)
C23A—C24A	1.373 (2)	C23B—H23B	0.9300
C23A—H23A	0.9300	C24B—H24B	0.9300
C24A—H24A	0.9300	O25—H25A	0.86 (2)
N1B—C5B	1.3120 (18)	O25—H25B	0.94 (3)
			
C5A—N1A—N2A	108.07 (11)	C3B—N2B—N1B	107.22 (11)
C3A—N2A—N1A	107.22 (11)	N2B—C3B—N4B	110.65 (12)
N2A—C3A—N4A	110.36 (12)	N2B—C3B—C6B	125.87 (13)
N2A—C3A—C6A	124.65 (13)	N4B—C3B—C6B	123.48 (12)
N4A—C3A—C6A	124.93 (12)	C3B—N4B—C5B	104.39 (11)
C3A—N4A—C5A	104.72 (11)	C3B—N4B—C12B	126.28 (11)
C3A—N4A—C12A	125.84 (11)	C5B—N4B—C12B	128.88 (11)
C5A—N4A—C12A	129.25 (11)	N1B—C5B—N4B	110.05 (12)
N1A—C5A—N4A	109.63 (12)	N1B—C5B—C19B	122.35 (12)
N1A—C5A—C19A	122.44 (12)	N4B—C5B—C19B	127.47 (12)
N4A—C5A—C19A	127.87 (12)	C3B—C6B—C7B	111.87 (12)
C3A—C6A—C7A	110.42 (11)	C3B—C6B—H6B1	109.2
C3A—C6A—H6A1	109.6	C7B—C6B—H6B1	109.2
C7A—C6A—H6A1	109.6	C3B—C6B—H6B2	109.2
C3A—C6A—H6A2	109.6	C7B—C6B—H6B2	109.2
C7A—C6A—H6A2	109.6	H6B1—C6B—H6B2	107.9
H6A1—C6A—H6A2	108.1	C8B—C7B—C9B	120.77 (14)
C8A—C7A—C9A	121.23 (14)	C8B—C7B—C6B	122.58 (14)
C8A—C7A—C6A	124.14 (14)	C9B—C7B—C6B	116.40 (13)
C9A—C7A—C6A	114.58 (12)	C7B—C8B—H8B1	120.0
C7A—C8A—H8A1	120.0	C7B—C8B—H8B2	120.0
C7A—C8A—H8A2	120.0	H8B1—C8B—H8B2	120.0
H8A1—C8A—H8A2	120.0	O10B—C9B—O11B	124.29 (13)
O10A—C9A—O11A	122.60 (13)	O10B—C9B—C7B	122.07 (13)
O10A—C9A—C7A	122.70 (13)	O11B—C9B—C7B	113.63 (12)
O11A—C9A—C7A	114.65 (12)	C9B—O11B—H11B	111.0 (13)
C9A—O11A—H11A	109.4 (16)	C13B—C12B—C17B	120.66 (13)
C17A—C12A—C13A	121.00 (13)	C13B—C12B—N4B	119.28 (13)
C17A—C12A—N4A	119.49 (12)	C17B—C12B—N4B	120.06 (13)
C13A—C12A—N4A	119.50 (12)	C12B—C13B—C14B	119.13 (14)
C12A—C13A—C14A	119.11 (14)	C12B—C13B—H13B	120.4
C12A—C13A—H13A	120.4	C14B—C13B—H13B	120.4
C14A—C13A—H13A	120.4	C15B—C14B—C13B	121.37 (15)
C15A—C14A—C13A	121.16 (14)	C15B—C14B—H14B	119.3
C15A—C14A—H14A	119.4	C13B—C14B—H14B	119.3
C13A—C14A—H14A	119.4	C14B—C15B—C16B	118.32 (14)
C14A—C15A—C16A	118.25 (14)	C14B—C15B—C18B	121.06 (17)
C14A—C15A—C18A	121.34 (14)	C16B—C15B—C18B	120.60 (17)
C16A—C15A—C18A	120.40 (14)	C17B—C16B—C15B	121.08 (15)
C17A—C16A—C15A	121.42 (14)	C17B—C16B—H16B	119.5
C17A—C16A—H16A	119.3	C15B—C16B—H16B	119.5
C15A—C16A—H16A	119.3	C12B—C17B—C16B	119.43 (15)
C12A—C17A—C16A	119.03 (14)	C12B—C17B—H17B	120.3
C12A—C17A—H17A	120.5	C16B—C17B—H17B	120.3
C16A—C17A—H17A	120.5	C15B—C18B—H18D	109.5
C15A—C18A—H18A	109.5	C15B—C18B—H18E	109.5
C15A—C18A—H18B	109.5	H18D—C18B—H18E	109.5
H18A—C18A—H18B	109.5	C15B—C18B—H18F	109.5
C15A—C18A—H18C	109.5	H18D—C18B—H18F	109.5
H18A—C18A—H18C	109.5	H18E—C18B—H18F	109.5
H18B—C18A—H18C	109.5	C20B—C19B—C24B	117.81 (13)
C20A—C19A—C24A	117.60 (13)	C20B—C19B—C5B	123.66 (12)
C20A—C19A—C5A	124.74 (12)	C24B—C19B—C5B	118.32 (12)
C24A—C19A—C5A	117.66 (12)	C21B—C20B—C19B	118.73 (14)
C21A—C20A—C19A	118.89 (13)	C21B—C20B—H20B	120.6
C21A—C20A—H20A	120.6	C19B—C20B—H20B	120.6
C19A—C20A—H20A	120.6	N22B—C21B—C20B	123.66 (14)
N22A—C21A—C20A	123.72 (14)	N22B—C21B—H21B	118.2
N22A—C21A—H21A	118.1	C20B—C21B—H21B	118.2
C20A—C21A—H21A	118.1	C21B—N22B—C23B	117.17 (13)
C23A—N22A—C21A	116.99 (13)	N22B—C23B—C24B	123.65 (14)
N22A—C23A—C24A	123.75 (13)	N22B—C23B—H23B	118.2
N22A—C23A—H23A	118.1	C24B—C23B—H23B	118.2
C24A—C23A—H23A	118.1	C23B—C24B—C19B	118.93 (14)
C23A—C24A—C19A	119.05 (13)	C23B—C24B—H24B	120.5
C23A—C24A—H24A	120.5	C19B—C24B—H24B	120.5
C19A—C24A—H24A	120.5	H25A—O25—H25B	107 (2)
C5B—N1B—N2B	107.69 (11)		
			
C5A—N1A—N2A—C3A	0.04 (16)	C5B—N1B—N2B—C3B	0.47 (16)
N1A—N2A—C3A—N4A	−0.71 (16)	N1B—N2B—C3B—N4B	−0.51 (16)
N1A—N2A—C3A—C6A	176.50 (13)	N1B—N2B—C3B—C6B	179.78 (13)
N2A—C3A—N4A—C5A	1.06 (15)	N2B—C3B—N4B—C5B	0.36 (16)
C6A—C3A—N4A—C5A	−176.14 (13)	C6B—C3B—N4B—C5B	−179.92 (13)
N2A—C3A—N4A—C12A	176.44 (12)	N2B—C3B—N4B—C12B	173.18 (13)
C6A—C3A—N4A—C12A	−0.8 (2)	C6B—C3B—N4B—C12B	−7.1 (2)
N2A—N1A—C5A—N4A	0.63 (16)	N2B—N1B—C5B—N4B	−0.25 (16)
N2A—N1A—C5A—C19A	−176.64 (12)	N2B—N1B—C5B—C19B	175.94 (12)
C3A—N4A—C5A—N1A	−1.02 (15)	C3B—N4B—C5B—N1B	−0.05 (15)
C12A—N4A—C5A—N1A	−176.18 (13)	C12B—N4B—C5B—N1B	−172.62 (13)
C3A—N4A—C5A—C19A	176.06 (13)	C3B—N4B—C5B—C19B	−176.00 (13)
C12A—N4A—C5A—C19A	0.9 (2)	C12B—N4B—C5B—C19B	11.4 (2)
N2A—C3A—C6A—C7A	−47.08 (19)	N2B—C3B—C6B—C7B	−7.6 (2)
N4A—C3A—C6A—C7A	129.72 (14)	N4B—C3B—C6B—C7B	172.73 (13)
C3A—C6A—C7A—C8A	113.07 (16)	C3B—C6B—C7B—C8B	118.41 (16)
C3A—C6A—C7A—C9A	−64.30 (15)	C3B—C6B—C7B—C9B	−67.30 (17)
C8A—C7A—C9A—O10A	−175.09 (15)	C8B—C7B—C9B—O10B	158.02 (15)
C6A—C7A—C9A—O10A	2.37 (19)	C6B—C7B—C9B—O10B	−16.4 (2)
C8A—C7A—C9A—O11A	2.28 (19)	C8B—C7B—C9B—O11B	−21.1 (2)
C6A—C7A—C9A—O11A	179.74 (11)	C6B—C7B—C9B—O11B	164.47 (12)
C3A—N4A—C12A—C17A	−86.66 (17)	C3B—N4B—C12B—C13B	−93.89 (17)
C5A—N4A—C12A—C17A	87.56 (18)	C5B—N4B—C12B—C13B	77.17 (19)
C3A—N4A—C12A—C13A	92.27 (17)	C3B—N4B—C12B—C17B	86.56 (18)
C5A—N4A—C12A—C13A	−93.51 (17)	C5B—N4B—C12B—C17B	−102.38 (17)
C17A—C12A—C13A—C14A	−1.0 (2)	C17B—C12B—C13B—C14B	0.4 (2)
N4A—C12A—C13A—C14A	−179.92 (13)	N4B—C12B—C13B—C14B	−179.14 (13)
C12A—C13A—C14A—C15A	1.4 (2)	C12B—C13B—C14B—C15B	0.5 (2)
C13A—C14A—C15A—C16A	−0.9 (2)	C13B—C14B—C15B—C16B	−0.5 (2)
C13A—C14A—C15A—C18A	179.02 (15)	C13B—C14B—C15B—C18B	178.25 (15)
C14A—C15A—C16A—C17A	0.0 (2)	C14B—C15B—C16B—C17B	−0.3 (2)
C18A—C15A—C16A—C17A	−179.95 (15)	C18B—C15B—C16B—C17B	−179.07 (16)
C13A—C12A—C17A—C16A	0.1 (2)	C13B—C12B—C17B—C16B	−1.2 (2)
N4A—C12A—C17A—C16A	179.02 (13)	N4B—C12B—C17B—C16B	178.34 (13)
C15A—C16A—C17A—C12A	0.4 (2)	C15B—C16B—C17B—C12B	1.2 (2)
N1A—C5A—C19A—C20A	−159.06 (14)	N1B—C5B—C19B—C20B	−151.82 (15)
N4A—C5A—C19A—C20A	24.2 (2)	N4B—C5B—C19B—C20B	23.7 (2)
N1A—C5A—C19A—C24A	20.3 (2)	N1B—C5B—C19B—C24B	22.8 (2)
N4A—C5A—C19A—C24A	−156.40 (14)	N4B—C5B—C19B—C24B	−161.69 (14)
C24A—C19A—C20A—C21A	0.1 (2)	C24B—C19B—C20B—C21B	−1.8 (2)
C5A—C19A—C20A—C21A	179.52 (14)	C5B—C19B—C20B—C21B	172.90 (14)
C19A—C20A—C21A—N22A	0.5 (2)	C19B—C20B—C21B—N22B	−0.3 (2)
C20A—C21A—N22A—C23A	−0.8 (2)	C20B—C21B—N22B—C23B	1.6 (2)
C21A—N22A—C23A—C24A	0.5 (2)	C21B—N22B—C23B—C24B	−0.8 (2)
N22A—C23A—C24A—C19A	0.1 (2)	N22B—C23B—C24B—C19B	−1.3 (2)
C20A—C19A—C24A—C23A	−0.4 (2)	C20B—C19B—C24B—C23B	2.5 (2)
C5A—C19A—C24A—C23A	−179.87 (13)	C5B—C19B—C24B—C23B	−172.47 (13)

### Hydrogen-bond geometry (Å, º)

**Table:**

*D*—H···*A*	*D*—H	H···*A*	*D*···*A*	*D*—H···*A*
O11*A*—H11*A*···N22*A*^i^	0.92 (3)	1.77 (3)	2.6838 (17)	178 (2)
O11*B*—H11*B*···O25	0.98 (2)	1.59 (2)	2.5619 (16)	174 (2)
O25—H25*A*···N1*A*	0.86 (2)	2.00 (2)	2.8373 (17)	166 (2)
O25—H25*B*···N22*B*^ii^	0.94 (2)	1.87 (2)	2.8050 (18)	171 (2)
C6*A*—H6*A*2···O10*B*^iii^	0.97	2.51	3.3300 (18)	142
C24*A*—H24*A*···O10*B*^iv^	0.93	2.41	3.2683 (18)	153
C24*B*—H24*B*···O10*A*^iii^	0.93	2.57	3.4628 (18)	162

Symmetry codes: (i) −*x*+1/2, *y*−1/2, −*z*+1/2; (ii) −*x*+1/2, *y*−1/2, −*z*+3/2; (iii) −*x*, −*y*+1, −*z*+1; (iv) −*x*+1, −*y*+1, −*z*+1.
